# Effect of aging and curing mode on the compressive and indirect tensile strength of resin composite cements

**DOI:** 10.1186/s13005-017-0155-z

**Published:** 2017-11-21

**Authors:** Nadja Rohr, Jens Fischer

**Affiliations:** Division of Materials Science and Engineering, Clinic for Reconstructive Dentistry and Temporomandibular Disorders, University Center for Dental Medicine, Hebelstrasse 3, CH-4056 Basel, Switzerland

**Keywords:** Resin composite cement, Indirect tensile strength, Compressive strength, Thermocycling, Self-adhesive cement

## Abstract

**Background:**

Resin composite cements are used in dentistry to bond ceramic restorations to the tooth structure. In the oral cavity these cements are subjected to aging induced by masticatory and thermal stresses. Thermal cycling between 5 and 55 °C simulates the effect of varying temperatures in vitro. Purpose of this study was to compare indirect tensile to compressive strength of different cements before and after thermal cycling. The effect of the curing mode was additionally assessed.

**Methods:**

Indirect tensile strength and compressive strength of 7 dual-curing resin composite cements (Multilink Automix, Multilink SpeedCem, RelyX Ultimate, RelyX Unicem 2 Automix, Panavia V5, Panavia SA Plus, Harvard Implant semi-permanent) was measured. The specimens were either autopolymerized or light-cured (*n* = 10). The mechanical properties were assessed after 24 h water storage at 37 °C and after aging (20,000 thermo cycles) with previous 24 h water storage at 37 °C.

**Results:**

Indirect tensile strength ranged from 5.2 ± 0.8 to 55.3 ± 4.2 MPa, compressive strength from 35.8 ± 1.8 MPa to 343.8 ± 19.6 MPa.

**Conclusions:**

Thermocyclic aging of 20,000 cycles can be considered a suitable method to simulate the degradation of indirect tensile strength but not compressive strength of resin composite cements. The effect of thermocycling and the curing mode on the resin composite cements is material dependent and cannot be generalized.

## Background

The use of esthetic ceramic materials in dentistry requires the application of resin composite cement to bond a restoration to the tooth structure. Resin composite materials are generally superior to conventional cements in providing higher strength, lower cement wear and improved esthetics [1–4]. Resin composite cements consist of three components: a polymer matrix, fillers and silanes that connect organic and inorganic phase [5–8]. These single components and their respective microstructure define the properties of the resin composite cement such as elasticity, hardness, strength and thermal as well as chemical stability [6, 9, 10]. To bond to the tooth substance, adhesive resin composite cements require the application of an acidic agent plus a priming system. Self-adhesive resin composite cements were thus designed to adhere to the tooth structure by themselves, while eliminating the need for additional pre-treatments of tooth structures. The polymer matrix of these self-adhesive resin cements is generally composed of phosphoric and/or carboxylic acid methacrylate monomers [3]. Self-adhesive cements interact only superficially with mineralized tissues hence they do not form a dentin hybrid layer nor resin tags [11, 12], resulting in lower bond strengths to both, dentin and enamel when compared to adhesive resin composite cements where an additional tooth conditioning system is applied [13]. Superior vickers hardness, modulus of elasticity, compressive and flexural strength were measured for adhesive cements in comparison to self-adhesive cements [3, 14].

The polymerization of dual-curing resin composite cements is catalyzed by a chemically (autopolymerization) and a photo (light-curing) activated initiator. The polymerization reaction starts with the mixing of base and catalyst paste, thus activating the chemical initiator. Hence the processing time is limited. Photo initiation allows to advance the polymerization reaction at the time a restoration is correctly placed and cement excess is removed. However, areas under an opaque restoration that are not reached by the light may not polymerize as much as dual-cured areas. Most cement materials reveal a higher degree of conversion by dual-curing compared to autopolymerization [15–17]. The degree of conversion of autopolymerized cements is influenced by the concentration of monomer and catalyst as well as the ambient temperature [18–20]. Cements with a high degree of conversion also provide better mechanical properties [5, 16, 21].

Resin composite cements are brittle materials and therefore more susceptible to tensile loading than to compressive stress [22, 23]. Although, compressive strength of a cement is an important factor to predict a restoration’s resistance against masticatory forces [24–26]. Cements in an aqueous medium such as saliva are exposed to a long-term aging process, which might significantly compromise their mechanical properties [27, 28]. The effects are wide-ranging but generally include the leaching of unreacted compounds and the degradation of the polymer network [27, 29]. To artificially age dental materials, several methods such as cyclic loading, water storage, or thermal cycling are commonly used. Thermal cycling between 5 and 55 °C simulates the effect of varying temperatures present in the oral cavity due to hot or cold beverages [30, 31]. The suggested duration of thermal cycling ranges from 3000 to 100,000 cycles [32–37]. It is proposed that 10,000 cycles may represent 1 year of service [38]. After the placement of a restoration, the cement is setting at 37 °C and polymerizes for up to 24 h, hence during this time, thermal stress is rare. Therefore, to imitate the clinical situation, prior to artificial aging the specimens should be stored at 37 °C for 24 h [22].

The impact of thermal cycling on indirect tensile strength and compressive strength has been systematically assessed for only one cement and should be verified with additional cements [22]. Purpose of this study was therefore to compare indirect tensile to compressive strength of a temporary, three self-adhesive and three adhesive cements before and after thermal cycling. The effect of the curing mode was additionally assessed. Hypotheses were that adhesive cements achieve higher indirect tensile and compressive strength than self-adhesive cements and that thermocyclic aging significantly decreases indirect tensile and compressive strength of the cements.

## Methods

Indirect tensile strength (ITS) and compressive strength (CS) of 7 dual-curing resin composite cements were measured (Table 1). The specimens were either autopolymerized or light-cured. ITS and CS were measured after 24 h water storage at 37 °C and after 24 h water storage at 37 °C followed by thermocyclic loading. Cylindrical test specimens 3 mm in height and diameter (*n* = 10) were produced using a customized Teflon mold. The cement was filled into the respective cavities of the mold and kept in place with a plastic foil and a glass plate on each side. 10 specimens were produced for each group and either autopolymerized or light cured for 20 s from both sides (Elipar S10, 3 M ESPE, Seefeld, Germany). All specimens were then stored in 37 °C water for 24 h. Aging was performed for the respective specimens using a thermocycler (Thermocycler THE-1100, SD Mechatronik, Feldkirchen-Westerham, Germany). The specimens were immersed alternately in water baths of 5 and 55 °C, using a sieve for storage and transportation. The cycle duration was 1 min with a dwell time in each water bath of 20 s and a transfer time between baths of 10 s. 20,000 cycles within 14 days were performed to age the specimens.

**Table 1:** Cement material composition provided by the manufacturer

	Name	Manufacturer	Type	Monomers	Fillers	Initiators
MLA	Multilink Automix	Ivoclar Vivadent	Adhesive resin composite	Base paste: Bis-GMA, HEMA, 2-dimethylaminoethyl methacrylate Catalyst paste: ethyoxylated bisphenol A dimethacrylate, UDMA, HEMA	40 vol% - Barium glass - Ytterbium trifluoride - Spheroid mixed oxide Particle size: 0.25–3.0 μm	Dibenzoyl peroxide
MSC	Multilink Speed CEM	Ivoclar Vivadent	Self-adhesive resin composite	Base paste: UDMA, TEGDMA, polyethylene glycol dimethacrylate Catalyst paste: polyethylene glycol dimethacrylate, TEGDMA, Methacrylated phosphoric acid ester, UDMA	40 vol% - Barium glass - Ytterbium trifluoride Particle size: 0.1–7 μm	Dibenzoyl peroxide
RUL	RelyX Ultimate	3 M ESPE	Adhesive resin composite	Base paste: methacrylate monomers containing phosphoric acid groups, methacrylate monomers Catalyst paste: methacrylate monomers	43 vol% - Silanated fillers - Alkaline (basic) fillers Particle size: 13 μm	Sodium toluene-4-sulphinate, Disodium peroxodisulphate, Tert-butyl 3,5,5-trimethylperoxyhexanoate
RUN	RelyX Unicem 2 Automix	3 M ESPE	Self-adhesive resin composite	Base paste: phosphoric acid modified methacrylate monomers, bi-functional methacrylate Catalyst paste: methacrylate monomers	43 vol% - Alkaline (basic) fillers - Silanated fillers Particle size: 12.5 μm	Sodium toluene-4-sulphinate, Sodium Persulfate, Tert-butyl 3,5,5-trimethylperoxyhexanoate
PV5	Panavia V5	Kuraray	Adhesive resin composite	Paste A: Bis-GMA, TEGDMA, Hydrophobic aromatic dimethacrylate, Hydrophilic aliphatic dimethacrylate Paste B: Bis-GMA, Hydrophobic aromatic dimethacrylate, Hydrophilic aliphatic dimethacrylate	38 vol% - Silanated barium glass filler - Silanated fluoroalminosilicate glass filler - Colloidal silica - Silanated alminium oxide filler Particle size: 0.01–12 μm	dl-Camphorquinone
PSA	Panavia SA plus	Kuraray	Self-adhesive resin composite	Paste A: 10-MDP, Bis-GMA, TEGDMA, Hydrophobic aromatic dimethacrylate, HEMA Paste B: Hydrophobic aromatic dimethacrylate, hydrophobic aliphatic dimethacrylate	40 vol% - Silanated barium glass filler - Silanated colloidal silica Particle size: 0.02–20 μm	dl-Camphorquinone
HIS	Harvard Implant semi-permanent	Harvard Dental International	temporary resin cement	Methacrylates, zinc oxide	–	–

*10-MDP* 10-Methacryloyloxydecyl dihydrogen phosphate, *Bis-GMA* bisphenol A-glycidyl methacrylate, *HEMA* 2-hydroxyethyl methacrylate, *TEGDMA* triethyleneglycol dimethacrylate, *UDMA* urethane dimethacrylate

**Fig. 1:**
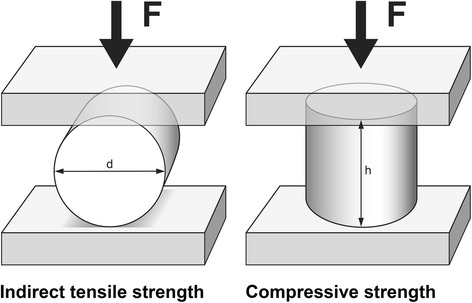
Test set-up for Indirect tensile and compressive strength (d = diameter, h = height, F = Force)

Specimens were loaded until fracture either after 24 h of water storage or after thermal cycling using a universal testing machine (Z020, Zwick/Roell, Ulm, Germany) (Fig. 1). Cross-head speed was set to 1 mm/min. Prior to the measurements, the specimens were sized in diameter and height using a digital caliper (Cal IP 67, Tesa, Ingersheim, Germany). For compressive strength the load was applied axially, for indirect tensile strength radially. Strength values were calculated using the following equations:

$$ Compressivestrength:{\sigma}_c=F/\pi {\left(d/2\right)}^2 $$

$$ Indirect tensile strength\ {\sigma}_t=2F/\pi dh $$

F is the fracture load; d the specimen diameter and h the specimen height. All data was tested for normal distribution using Shapiro-Wilk test. Since data was normal distributed, one-way ANOVA was applied followed by a Tukey HSD test to check for differences between the cement groups of ITS and (*p <* 0.05). Three-way ANOVA was performed with all ITS and CS values to test the effect of cement, curing mode and aging procedure (statplus pro V6.1.25, Analystsoft).

## Results

**Fig. 2:**
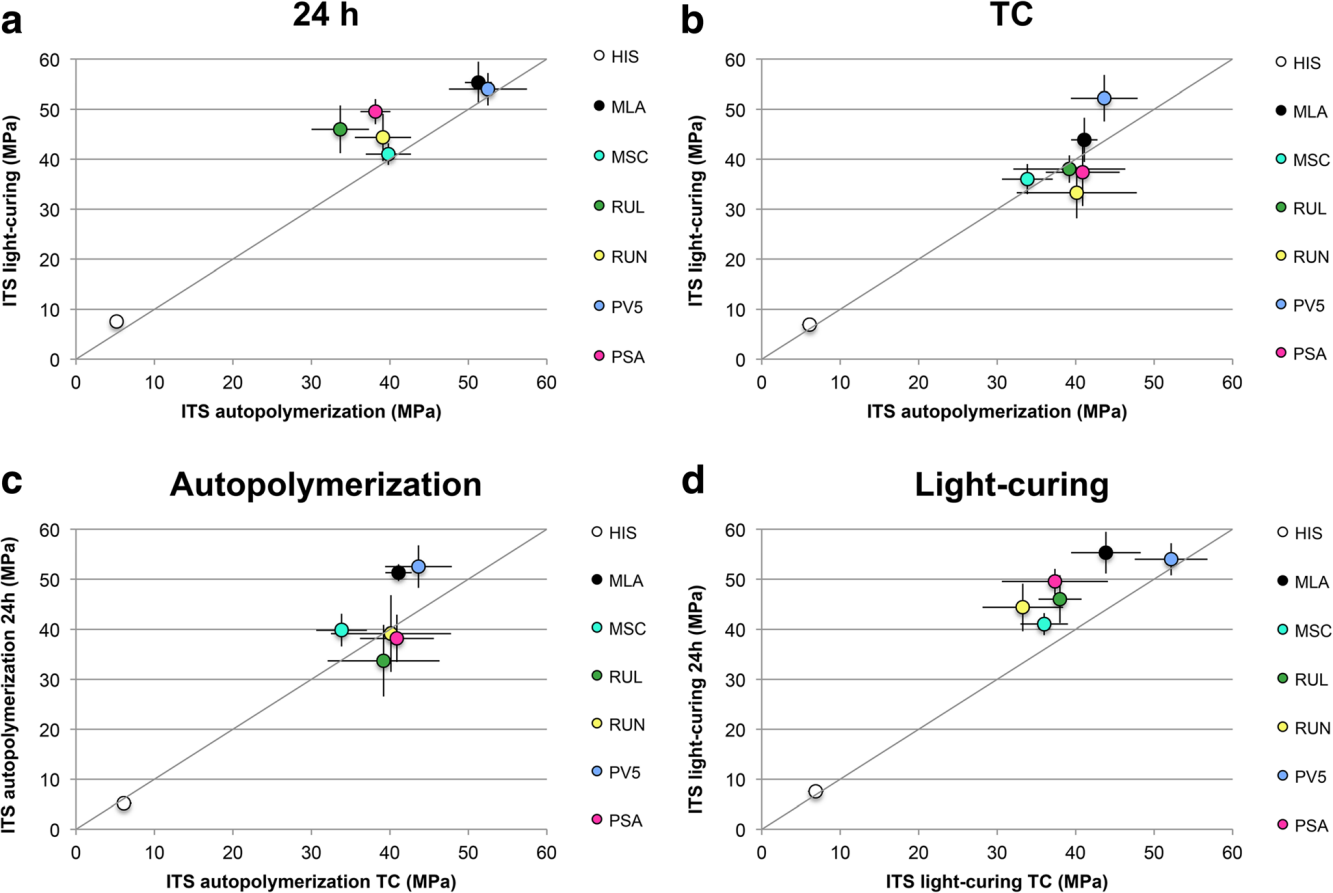
Indirect tensile strength (ITS) mean values of all cements. Values approaching the diagonal grey line indicate similar values on x-and y-axis **a** Comparison between light-cured and autopolymerized specimens after 24 h water storage at 37 °C **b** Comparison between light-cured and autopolymerized specimens after thermal cycling (TC) **c** Comparison between light-cured specimens after 24 h water storage at 37 °C and thermal cycling **d** Comparison between autopolymerized specimens after 24 h water storage at 37 °C and thermocyclic-aging

**Fig. 3:**
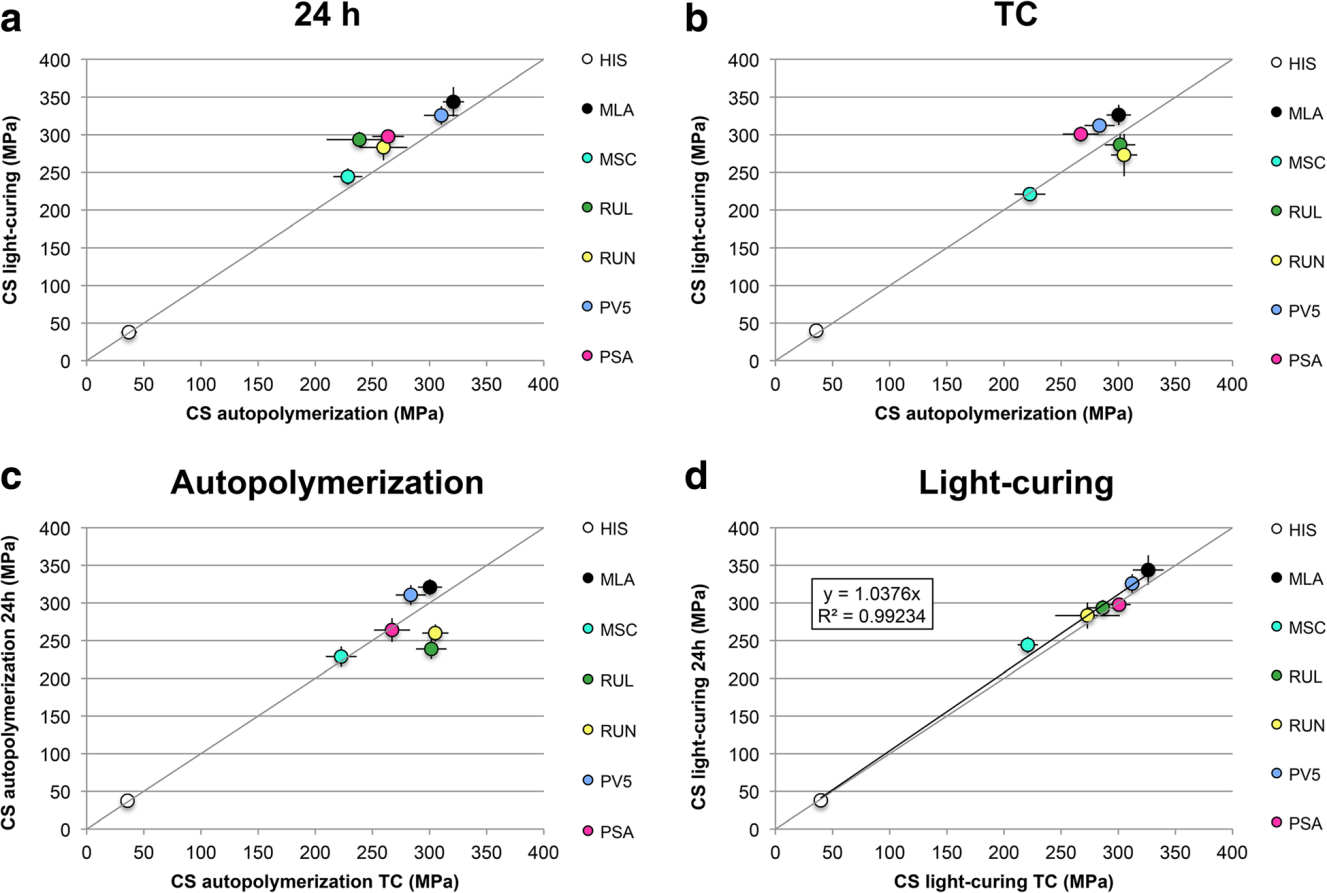
Compressive strength (CS) mean values of all cements. Values approaching the diagonal grey line indicate similar values on x-and y-axis **a** Comparison between light-cured and autopolymerized specimens after 24 h water storage at 37 °C **b** Comparison between light-cured and autopolymerized specimens after thermal cycling (TC) **c** Comparison between light-cured specimens after 24 h water storage at 37 °C and thermal cycling **d** Comparison between autopolymerized specimens after 24 h water storage at 37 °C and thermocyclic-aging

Values for ITS and CS are listed in Table 2. Values of ITS or CS with no statistical difference within one cement are marked with identical superscript letters. To visualize the effect of aging and curing mode on the different cements, the mean values are correlated in Figs. 2 for ITS and Fig. 3 for CS. A grey line in each graphic indicates similar values on x-and y-axis meaning that if the dot of a material is close to the grey line, there is no effect of either a) curing-mode after 24 h, b) curing-mode after thermal cycling c) aging of light-cured specimens or d) aging of autopolymerized specimens.

**Table 2:** Indirect tensile strength and compressive strength mean values with standard deviations of the cements for light-cured and autopolymerized specimens after 24 h water storage at 37 °C (24 h) and aging (TC: 24 h water storage at 37 °C followed by 20,000 thermocycles)

(MPa)	Indirect tensile strength				Compressive strength			
	light-curing		autopolymerization		light-curing		autopolymerization	
cement	24 h	TC	24 h	TC	24 h	TC	24 h	TC
MLA	55.3 (4.2)^A^	43.9 (4.4)^B^	51.3 (1.7)^C^	41.1 (1.7)^B^	343.8 (19.6)^A^	326.3 (13.5)^B^	321.0 (9.3)^B^	300.5 (10.6)^C^
MSC	41.0 (2.2)^A^	36.0 (3.0)^B^	39.8 (2.9)^A^	33.9 (3.2)^B^	244.3 (11.0)^A^	220.9 (8.9)^B^	228.6 (12.7)^B^	222.9 (13.5)^B^
RUL	46.0 (4.8)^A^	38.0 (2.7)^B^	33.7 (3.7)^B^	39.2 (7.1)^B^	293.5 (10.5)^A^	286.6 (14.5)^A^	238.8 (28.8)^B^	301.7 (13.3)^A^
RUN	44.4 (4.7)^A^	33.3 (5.1)^B^	39.1 (3.6)^A,B^	40.2 (7.6)^A^	283.2 (17.3)^A,B^	273.1 (28.2)^A^	259.9 (20.8)^A^	305.2 (11.5)^B^
PV5	54.0 (3.2)^A^	52.2 (4.6)^A^	52.5 (5.0)^A^	43.7 (4.2)^B^	325.8 (12.3)^A^	312.3 (6.6)^A,B^	310.5 (15.2)^B^	283.8 (13.2)^C^
PSA	49.5 (2.5)^A^	37.4 (6.8)^B^	38.2 (1.9)^B^	40.9 (4.7)^B^	297.8 (7.1)^A^	300.8 (10.1)^A^	263.9 (13.8)^B^	267.3 (15.8)^B^
HIS	7.5 (1.4)^A^	6.9 (1.0)^A,B^	5.2 (0.8)^C^	6.1 (1.0)^B,C^	37.7 (3.3)^A^	39.8 (1.8)^A^	37.1 (7.0)^A^	35.8 (1.8)^A^

Values of ITS or CS with no statistical difference within one cement are marked with superscript letters (horizontal comparison)

### Indirect tensile strength

ITS after 24 h water storage ranged within all groups between 5.2 ± 0.8 MPa for the autopolymerized temporary cement (HIS) and 55.3 ± 4.2 MPa for a light-cured adhesive resin composite cement (MLA). Effects of aging and light-curing mode on ITS are visualized in Fig. 2. Statistical higher (MLA, RUL, PSA and HIS) or values with no statistical difference (MSC, RUN, PV5) were obtained for light-cured specimens compared to autopolymerized specimens after 24 h water storage (Fig. 2a). When light-cured specimens were compared to autopolymerized specimens after thermo-cycling, values of RUN were significantly lower (*p* = 0.038) and of PV5 significantly higher (*p <* 0.001) (Fig. 2b). For autopolymerized specimens, aging in the thermocycler significantly decreased values of MLA, MSC and PV5 (Fig. 2c). No statistical different values were found for the other cements before and after aging of autopolymerized specimens. Aging of light-cured specimens significantly decreased ITS of MLA, MSC, RUL, RUN and PSA (Fig. 2d). Values for PV5, and HIS remained constant. Of all cements, highest values in all groups were obtained by either MLA or PV5. The ranking between MSC, RUL, RUN and PSA changed, depending on the curing or aging mode applied. HIS achieved statistically lowest values of all cements in all groups (*p <* 0.001). Three-way ANOVA revealed a significant effect on the ITS values of the cement, curing mode as well as the aging procedure (*p* < 0.001).

### Compressive strength

CS ranged between 35.8 ± 1.8 MPa for autopolymerized and aged HIS and 343.8 ± 19.6 MPa for light-cured MLA after 24 h water storage. Effects of aging and light-curing mode on CS are visualized in Fig. 3. For specimens after 24 h water storage, light-curing increased CS values significantly for MLA, MSC, RUL and PSA (Fig. 3a). After thermo-cycling, CS of light-cured specimens was significantly higher for MLA, PV5 and PSA (Fig. 2b). Autopolymerized specimens of RUN achieved significantly higher CS after aging than light-cured specimens (*p =* 0.006) (Fig. 3b). For autopolymerized specimens, aging significantly decreased CS for MLA and PV5 and increased CS of RUN and RUL. CS of all other cements (MSC, PSA, HIS) remained constant (Fig. 3c). Aging of light-cured specimens did not affect CS for all cements except MLA and MSC where the CS significantly dropped after aging. A linear correlation (y = 1.038×/R^2^ = 0.992) was found for CS before and after aging for light-cured specimens (Fig. 3d).

For light cured specimens cements ranked as follows before and after aging: MLA > PV5 > PSA > RUL > RUN > MSC > HIS. For autopolymerized specimens before aging ranking was similar to the light-cured except for RUL and RUN switching places. After aging the cements ranked: RUN > RUL > MLA > PV5 > PSA > MSC > HIS. Three-way ANOVA revealed a significant effect on the CS values of the cement and curing mode (*p* < 0.001), but not of the aging procedure (*p =* 0.709).

### Correlation between indirect tensile and compressive strength

**Fig. 4:**
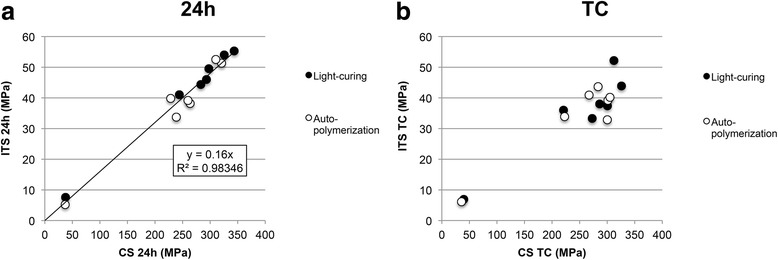
Comparison of indirect tensile (ITS) and compressive strength (CS) after **a** 24 h of water storage at 37 °C and **b** thermocyclic aging for autopolymerized and light-cured specimens

A linear correlation (y = 0.160×/R^2^ = 0.983) was found between ITS and CS for light-cured (y = 0.162×/R^2^ = 0.992) and autopolymerized (y = 0.158×/R^2^ = 0.960) specimens after 24 h of water storage (Fig. 4). After thermo-cycling cements were affected differently by the aging process as described above, hence ITS and CS did not correlate likewise.

## Discussion

Indirect tensile strength of a temporary, three self-adhesive and three adhesive cements was compared to compressive strength before and after thermal cycling. The effect of the curing mode was additionally assessed. The hypotheses that adhesive cements achieve higher indirect tensile and compressive strength than self-adhesive cements was rejected because the mechanical properties depended rather on the cement’s individual composition and filler types. That thermocyclic aging significantly decreases indirect tensile and compressive strength of the cements was verified for indirect tensile strength but not for compresssive strength.

### Indirect tensile strength

After 24 h water storage higher ITS values were recorded for all light-cured cements than for autopolymerized, although the difference was only significant for MLA, RUL, PSA and HIS. This difference was probably due to a higher degree of polymerisation of the light-cured specimens, as it was previously reported [17, 20–22, 36].

After thermo-cycling ITS value of light-cured RUN was significantly lower and of PV5 significantly higher than the values obtained after autopolymerization. Aging affected each cement differently, hence no distinct effect of the curing mechanism could be observed when autopolymerized and light-cured specimens were compared after aging.

Aging of autopolymerized specimens significantly decreased values of MLA, MSC and PV5. ITS of the other cements remained constant. The decrease of ITS of autopolymerized MLA, MSC and PV5 specimens indicates that these materials are more susceptible to temperature changes at the surface which may have induced the formation of superficial micro-cracks favored by the degradation of the polymer matrix and the absorption of water. An insufficient polymerization due to autopolymerization may have also resulted in a higher rate of unreacted and potentially leaching components inducing an increased surface inhomogeneity.

Aging of light-cured specimens significantly decreased ITS of MLA, MSC, RUL, RUN and PSA. Due to the high ITS of the light-cured specimens after 24 h, these specimens may also be more susceptive to aging than the autopolymerized specimens.

### Compressive strength

Higher CS values were obtained for light-cured specimens compared to autopolymerized specimens after 24 h water storage, although the difference was only statistically significant for MLA, MSC, RUL and PSA. These findings are consistent with the ones for ITS and due to the increased degree of conversion of the light-cured specimens. In comparison to the other cements, MLA, RUL and PSA revealed a stronger dependence on light-curing to achieve highest strength values. PSA contains 10-Methacryloyloxydecyl dihydrogen phosphate (MDP) inhibiting the polymerization reaction [39]. Significantly lower values were found for autopolymerized CS values of PSA compared to light-cured specimens after 24 h indicating that the polymerization reaction might have still been proceeding.

After thermo-cycling, CS of light-cured specimens was significantly higher for MLA, PV5 and PSA but lower for RUN compared to autopolymerized specimens. For RUN results were inverse, which might be explained by a higher amount of unreacted phosphoric acid ester groups, resulting in a higher degree of water up-take and thus an increased CS. Higher sorption was previously recorded for RUN for autopolymerized specimens [9]. Aging of autopolymerized specimens significantly decreased CS for MLA and PV5 due to a degradation of the material that might be due to a lower degree of polymerization than for the light-cured specimens. Values of RUN and RUL were increased after aging. RUL and RUN previously presented high sorption that might have been responsible for increasing their strength after thermal cycling [9]. Light-cured specimens correlated linearly before and after aging and were therefore less susceptible to aging than autoplymerized specimens. Since three-way ANOVA revealed no significant effect of the aging with 20,000 thermocycles on the CS values, the applied aging protocol does not seem suitable for this test method. Effects of a prolonged cycling should be further investigated.

### Correlation between indirect tensile and compressive strength

The filler content [7], the degree of conversion [23] and the monomer type [8] are factors affecting the mechanical strength of resin composite cements. Autopolymerized specimens revealed a stronger variability in CS and ITS than dual-cured specimens [15, 17, 22]. As previously reported the effect of the curing mode varied among the cements and cannot be generalized [36]. According to the linear correlation between CS of light-cured specimens before and after aging CS was less affected by aging than ITS indicating that the mechanical properties measured with a CS test are less susceptible to thermocyclic aging and for light-cured specimens the material properties are more stable, which findings are in accordance with previous results [22]. CS and ITS correlate linearly after 24 h water storage but not after aging because the cements age differently depending on their components.

### Test method

Previously it was reported that a compressive strength test is a rather insensitive test method compared to indirect tensile strength [22] or flexural strength [28]. In the present study it was found that both ITS and CS tests have their eligibility since aging of the cements resulted in different effects for either ITS or CS. The ITS test is more sensitive to surface defects than the CS test [22]. CS test may depend mainly on the filler size and distribution and the quality of silanization. It is probably also affected by the mode of polymerization. The effect of the degradation mechanism on the ITS and CS should be further investigated.

Thermocyclic aging has been evaluated as the most efficient aging procedure and was recommended to perform for at least 4 days for resin composite cement [22]. Thermal cycling has a considerable effect on the cements’ strength and the degree of the effect varied according to the cement’s composition. It is suggested that the temperature change and the associated dimensional changes of the two phases – polymer matrix and fillers – generate internal stress [22, 35, 37] due to different coefficients of thermal expansion of organic and inorganic fillers [22].

Thermocyclic aging has been previously applied to ITS and CS specimens of different resin composite cements but only for 2000 cycles within 20 h [36]. In that study no statistically significant differences between the values after aging were found. Therefore, a thermocycling duration of 2000 cycles within 20 h can be considered insufficient to successfully age resin composite cements. In the present study 20,000 cycles were performed within 14 days, providing measurable aging effects on ITS values.

### Clinical implications

CS test predicts the resistance against the masticatory force and therefore allows to estimate the cements clinical performance [24]. Materials with low intrinsic strengths such as silicate ceramics achieve a higher loading capacity when cemented with adhesive cement than with glass-ionomer [25]. A cement with a compressive strength above 320 MPa is ideal for cementing silicate ceramics on zirconia implants since the cement optimally supports the restorative material [26]. Since these 320 MPa were measured for autopolymerized cements after 24 h 37 °C water storage, in the present investigation PV5 and MLA can be considered best cements applying to this requirement with mean autopolymerized CS values of 312 and 326 MPa. Although after aging of the autopolymerized specimens, the cements do not exceed the 320 MPa.

According to ISO 9917–1:2007 for water based dental cements, CS of dental cements should be over 70 MPa. All cements except HIS fulfill this requirement. HIS is not indicated for permanent cementation but for a long-term temporary cementation on implants. When covered by bulky restorations cements might be insufficiently light-cured [21], which can also affect the mechanical strength of the cements. For most cements light-curing was beneficial to increase the mechanical strength. Only RUN and RUL revealed better or similar mechanical properties after thermocyclic aging of autopolymerized specimens than of light-cured ones, which may be explained by more intense water uptake.

## Conclusions

Within the limitations imposed by the current study, the following conclusions were drawn:

- Indirect tensile and compressive strength of the cements after 24 h water storage correlate linearly.
- Thermocyclic aging of 20,000 cycles can be considered a suitable method to simulate the degradation of indirect tensile strength but not compressive strength of resin composite cements.
- The effect of thermocycling on the resin composite cements is material dependent and cannot be generalized.

## Abbreviations

CS: Compressive strength
HIS: Harvard Implant semi-permanent
ITS: Indirect tensile strength
MLA: Multilink Automix
MSC: Multilink SpeedCem
PSA: Panavia SA plus
PV5: Panavia V5
RUL: RelyX Ultimate
RUN: RelyX Unicem 2 Automix
TC: Thermal cycling
